# The Clinical Presentation of Endometriosis and Its Association to Current Surgical Staging

**DOI:** 10.3390/jcm12072688

**Published:** 2023-04-04

**Authors:** Matilda Shaked Ashkenazi, Ole Linvåg Huseby, Gard Kroken, Marcela Trocha, Aurora Henriksson, Hanna Jasiak, Karen Cuartas, Alessandra Loschiavo, Isabella Kuhn, Dina Støve, Hanna Grindahl, Emilia Latour, Mathias Melbø, Katrine Holstad, Sebastian Kwiatkowski

**Affiliations:** 1 Department of Obstetrics and Gynaecology, Pomeranian Medical University in Szcezcin, 70-204 Szczecin, Poland; 2 Fiskeridirektoratet, Strandgaten, 5004 Bergen, Norway; 3 Department of Obstetrics and Gynaecology, Poznan University of Medical Sciences, 61-701 Poznan, Poland; 4 Department of Obstetrics and Gynaecology, University of Texas Southwestern Medical Center, Dallas, TX 75390, USA; 5 The Department of Woman, Child, and General and Specialized Surgery, Università della Campania Luigi Vanvitelli, 81100 Caserta, Italy; 6 Musculoskeletal University Center Munich, Department of Orthopaedics and Trauma Surgery, University Hospital, LMU Munich, 80336 Munich, Germany

**Keywords:** endometriosis, pelvic pain, fertility, quality of life

## Abstract

(1) Despite its high prevalence, the diagnostic delay of endometriosis is still estimated to be about 7 years. The objective of the present study is to understand the symptomatology of endometriosis in patients across various countries and to assess whether the severity of symptoms correlates with the diagnosed stage of disease. (2) An international online survey collected self-reported responses from 2964 participants from 59 countries. Finalization of the questionnaire and its distribution was achieved by cooperation with various organizations and centers around the globe. (3) Chronic pain presentation remarkably increased between Stage 1 and 2 (16.2% and 32.2%, respectively). The prevalence of pain only around and during menstruation was negatively correlated to the stage, presenting with 15.4% and 6.9% in Stages 1 and 4, respectively. Atypical presentation of pain was most commonly reported in stage 4 (11.4%). Pain related solely to triggering factors was the most uncommon presentation of pain (3.2%). (4) Characteristics of pain and quality of life tend to differ depending on the reported stage of the endometriosis. Further research may allow a better stage estimation and identification of patients with alarming symptomatic presentation indicative of a progressive stage, even those that are not yet laparoscopically diagnosed.

## 1. Introduction

Endometriosis is a chronic, inflammatory disease characterized by the presence of functional endometrial tissue outside of the uterine cavity, most commonly involving the ovaries. It is one of the most common gynecological diseases among all ethnicities and social groups [1], and affects about 10% of women of reproductive age [2]. The main clinical manifestations are chronic pelvic pain and infertility [3]. Despite its prevalence, endometriosis is still under-diagnosed, under-researched and under-reported, and therefore further research into its impact on various factors is critical [4].

Among the symptoms presented in endometriosis, varying manifestations of pain are one of the most prominent. Pain constitutes the three frequently primary symptoms: dysmenorrhoea, lower abdominal pain, and pelvic pain [5]. Deep dyspareunia, or pain with sexual intercourse, is also considered a fourth primary symptom [6]. Other frequently reported symptoms include abdominal pain on urination, lower back pain, pain on defecation where hemorrhoids are excluded, and cyclical extrapelvic pain [5]. While pain has been identified in the progression of the disease, there is still a lack of research on the link between pain and other symptoms of endometriosis that are specific to the stage of disease.

Additional major symptoms associated with endometriosis include impaired fertility, which affects up to 50% of patients [7].

Endometriosis also impairs patients’ mental and physical well-being and has a significant impact on patients’ quality of life [8]. Furthermore, previous literature has demonstrated clearly that endometriosis decreases patients’ education and work productivity, mental health, and well-being [8].

This study aimed to explore the relationship between the clinical presentation of endometriosis, namely pain and quality of life, to the current surgical staging of the disease. Given that there are currently no clear unified guidelines on placing patients with alarming symptoms into later stages of the disease, the intent of this study is to identify such symptoms with the aim of identifying patients who will most likely benefit primarily from surgical and/or fertility treatments. The prioritization of such patients for these treatments will help to avoid the possible delays associated with the wait for laparoscopic diagnosis and the initiation of hormonal treatments that are currently the first line of treatment offered for patients without definitive (laparoscopic) diagnosis.

## 2. Materials and Methods

An international cross-sectional survey was employed for this study, created along an initial qualitative phase that consisted of a scoping literature review, created with the purpose of identifying globally accepted characteristics of pain and related symptoms of endometriosis. A computerized search of PubMed Central-US National Library of Medicine (PMC), the Biomed Central Women Health (BMC), coherence library, and Health Affairs resources was performed to identify relevant articles. Data was pulled from articles about endometriosis and its management in the field of obstetrics and gynecology, focusing on the different symptomatic manifestations of the condition as well as its impact on quality of life and healthcare management. The search was conducted using the following terms: ‘Endometriosis and quality of life’; ‘endometriosis and infertility’; ‘endometriosis and pain’; and ‘endometriosis and symptoms’.

Information from qualitative studies, multicentre studies, comparative studies, controlled and randomized controlled trials, and clinical trials was included in the resulting literature review. The following inclusion criteria were used to choose the articles: they had to have been published within the last five years, be written in English and published in peer-reviewed journals, and had to involve questionnaire studies on endometriosis consisting of self-reported surveys. The results of the review will not be further explored in this manuscript, as the qualitative phase did not produce the analysis that is necessary for the interpretation of the findings.

### 2.1. Translation

The survey was created using a methodologic approach in which questions aimed to investigate selected factors that may or may not affect women’s suffering from endometriosis and their quality of life. The initial survey was first finalized in English and later translated into 15 languages, which included Arabic, Farsi, Finnish, French, German, Greek, Hebrew, Italian, Norwegian, Polish, Portuguese, Russian, Spanish, Swedish, and Turkish, in accordance with the World Health Organization (WHO) recommendations for medical translation. Translations were aimed at the conceptual equivalent of relevant phrases and words, as recommended by the WHO (World Health Organization, 2017), yet avoided a ‘word-for-word’ or literal translation. It aimed for all three phases of forward translation, expert panel, and back translation for every language. Distributed through different platforms, this survey provided data from 2964 respondents with a self-reported endometriosis diagnosis.

### 2.2. Questionnaire Structure 

The questionnaire was divided into four sections. The first section included an informed consent paragraph and collected demographic information from respondents, including questions on age, nationality and country of residence. The second section investigated the health-related quality of life (HRQoL) in women with endometriosis. Based on a review of EHP-30 [9] questions about pain experience, the effect on everyday life and the need for medical attention were also included. In questions related to pain experience, those about menstrual cycles and sexual intercourse were taken into consideration, although limitation of activity responses were evaluated in the section regarding impact on everyday life. This section also included questions about disease stage and time of diagnosis, as well as current treatment approaches and fertility treatments.

The third and fourth sections of the questionnaire focused on the effects of the COVID-19 pandemic on women with endometriosis. These parts were excluded in the present article and are discussed more thoroughly in a different paper [10].

For the purpose of analyzing the results, the research team created questions that enabled the division of participants into different groups that were later co-analyzed with their stage and additional factors. The questionnaire was validated for content and construct, reviewed by an expert panel, and piloted on endometriosis patients before administration.

The questionnaire, being an online self-administered survey, was distributed using social media channels provided by the cooperating organizations and centers. The participants reported back voluntarily, without any form of compensation. Because the study did not employ pre-existing databases of patient histories, the respondents were not recruited at health care centers. The material collected from these self-reported data was subsequently interpreted for further analysis. Surgical diagnosis based on ASRM staging was a selective criterion for patients reporting to be diagnosed with a specific stage.

### 2.3. Group Characteristics

Patients participating in the study were divided into five groups according to their diagnosis based on the ASRM (The American Society of Reproductive Medicine) classification: Stage 1; Stage 2; Stage 3; Stage 4, and undiagnosed patients. Due to the international nature of this study, the research team aimed to employ a criterion that is used internationally and that patients are familiar with. As the ASRM criteria has been accepted globally and has been widely used in recent years, it was therefore selected [11]. An analysis of pain complaints in addition to the impact of pain on quality of life and fertility was then carried out for each group.

The effect on quality of life was classified as: (A): severe long-term effect; (B): severe short term effect; (C): mild effect and (D): no effect. Different manifestations of pain were also investigated and groupings were created; (1): chronic pain throughout the menstrual cycle (2): pain only around and during menstruation, (3): pain related to trigger factors, (4): pain related only to sexual intercourse, (5): mixed presentation of pain (selected multiple options), (6): other experiences of pain, and (7): little to no experience of pain.

### 2.4. Ethical Approval

The Bioethics Committee of the Pomeranian Medical University in Szczecin provided an exemption from ethical consent (case number: KB-0012/34/03/2021/Z). Additionally, this study was granted an ethical approval from the Turkish Ministry of Health (2021-01-13T17_02_26, Başvuru Formu için tıklayınız//KONU No: KAEK/2021.0ƒ1.27).

### 2.5. Data Analysis

**Figure 1:**
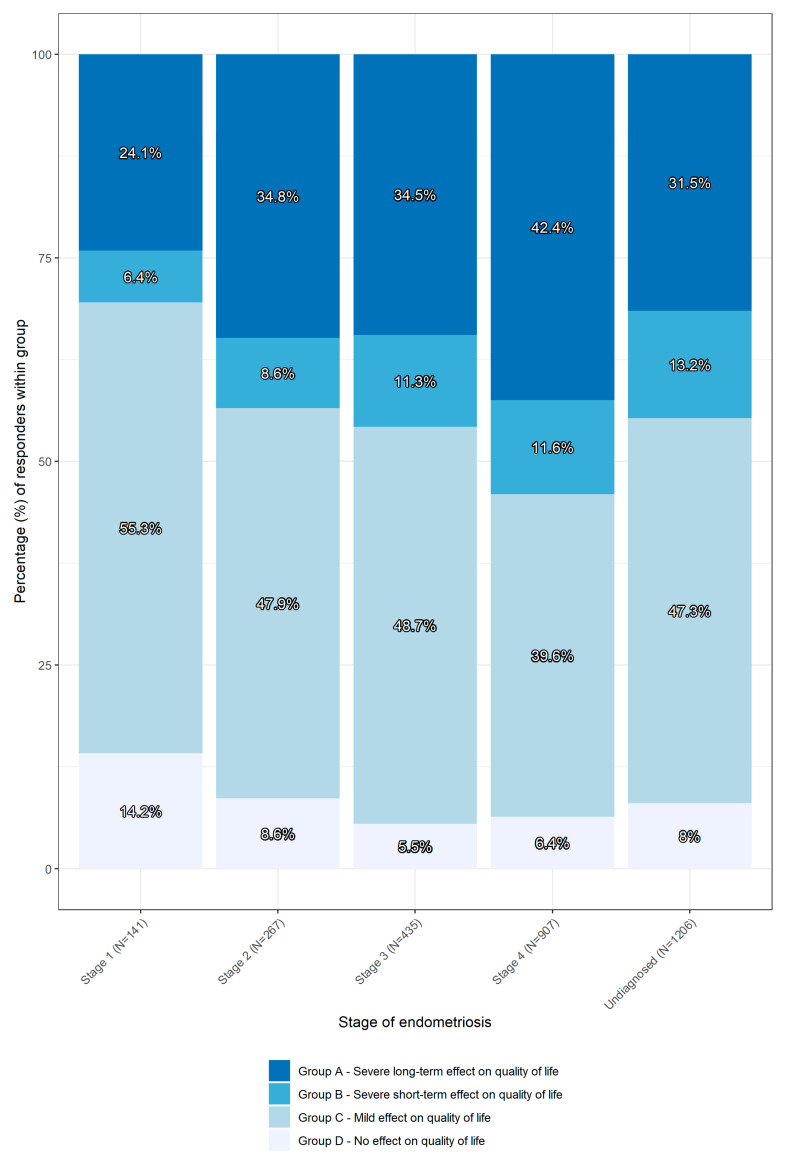
Reported everyday experiences of quality of life in relation to the stage. The figure shows the percentage of participants within a defined group related to the reported changes in quality of life. The percentages of participants are shaded. Group A is represented by the darkest shade, while group D is represented by the brightest shade.

**Figure 2:**
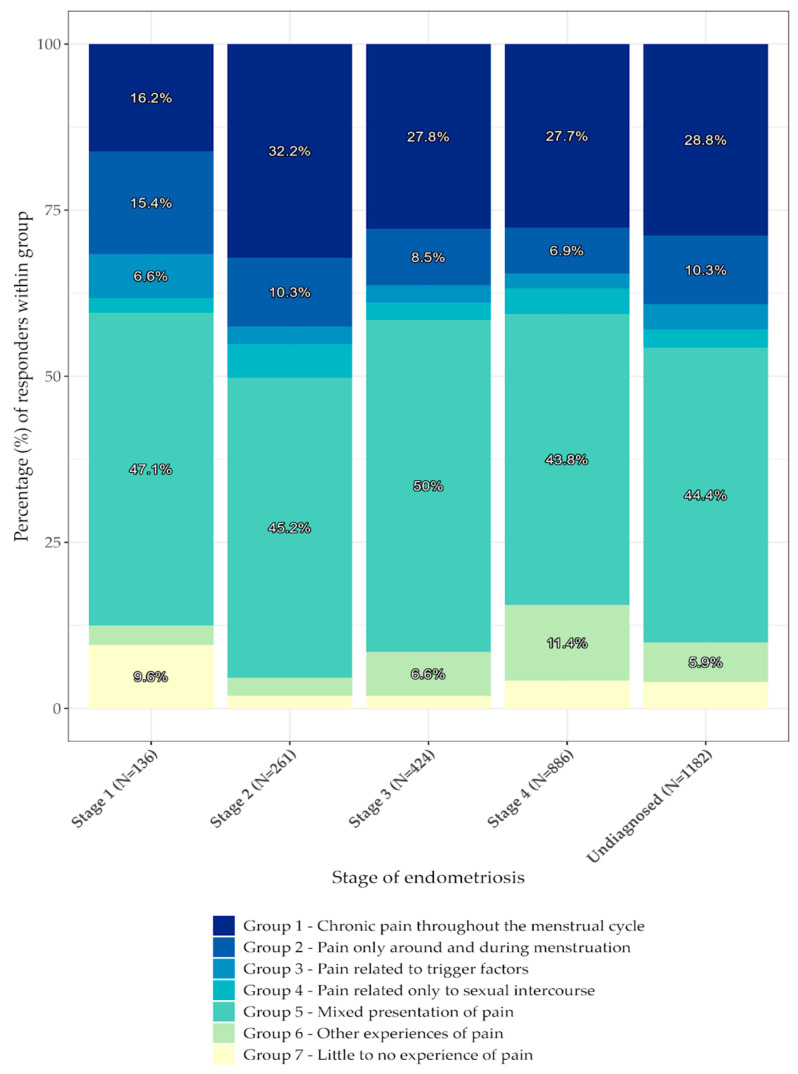
Reported experiences of pain in relation to stage. The figure shows the percentage of participants within a defined group and their reported pain related to the stages of disease. The percentages of respondents are shaded. The darkest shade represents group 1, and gets brighter until group 7, which is represented by the brightest shade.

**Figure 3:**
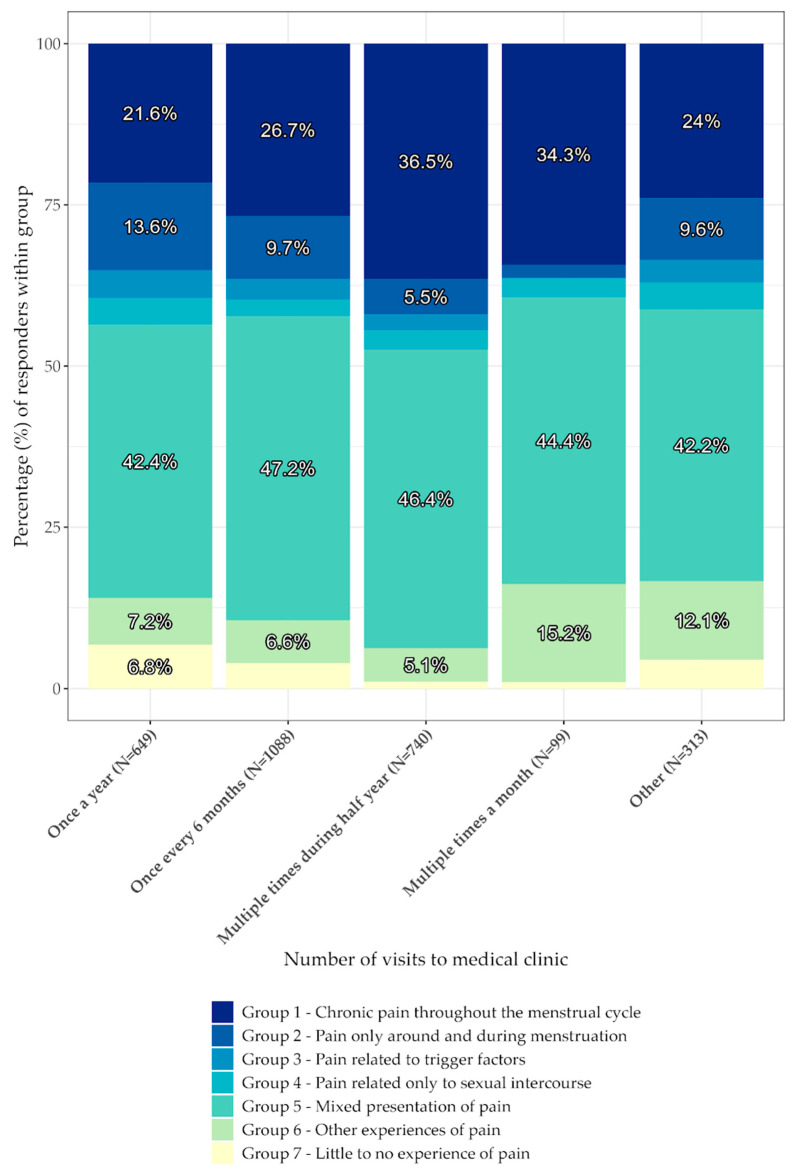
Reported pain in relation to medical appointments. The figure shows the percentage of participants within a defined group related to the frequency of their medical appointments. The percentages of respondents are shaded accordingly; group 1 is represented with the darkest shade (in blue), while group 7 is represented with the brightest shade (in yellow).

**Figure 4:**
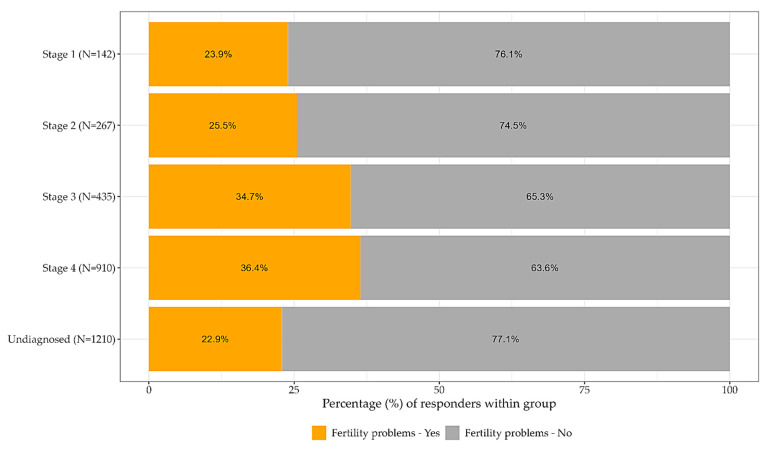
Fertility abnormalities in relation to stage. The figure shows the percentage of participants who experience fertility abnormalities in relation to their reported stages. The percentages of respondents are shaded according to their answer.

The data were analyzed and plotted using R (R: The R Project for Statistical Computing, Vienna, Austria). Chi-square tests for independence were carried out for everyday experience and stage, pain experience and stage, and pain experience and the frequency of visits to medical institutions. *p* values of <0.05 were considered nominally significant (shown in Figure 1, Figure 2, Figure 3 and Figure 4).

**Figure 5:**
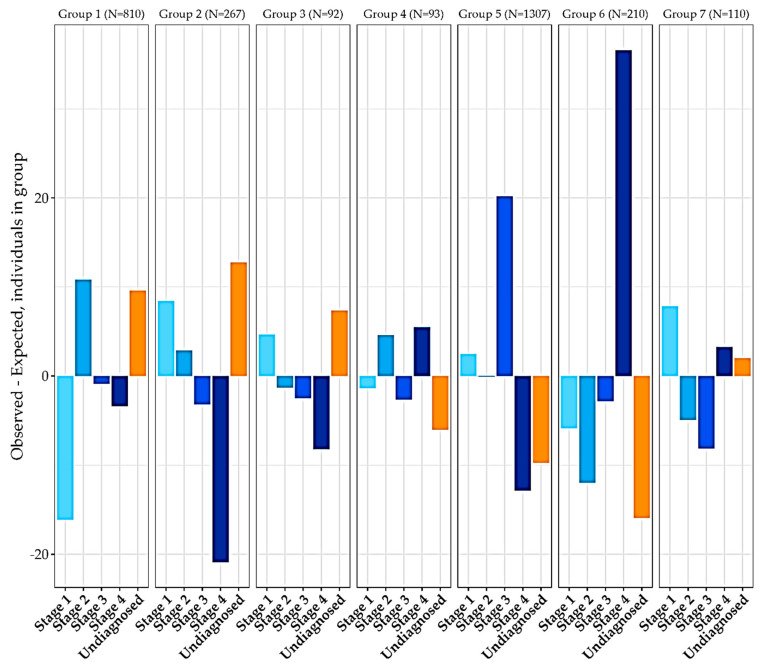
Observed distribution of pain minus expected distribution of pain in relation to stage. The figure shows the number of individuals in a defined group and the observed distribution of pain minus the expected distribution of pain in relation to their reported stages. The various stages are shaded. The brightest shade represents Stage 1, while the darkest shade represents Stage 4. The column to the right of “Stage 4” represents participants who were not diagnosed with a specific stage at the time of the completion of the questionnaire.

For added clarity into the correlation of pain and stage of endometriosis, another graph was plotted (Figure 5) showing the observed responses against the expected results in the absence of correlation. The expected values were initially calculated by detecting the ratio of different pain categories in the population. The data was subsequently divided into groups by endometriosis stage. Next, the number of participants in each pain category within these groups was multiplied by the ratio of the ungrouped pain categories, achieving the expected number of participants with the given pain category and stage.

## 3. Results

Out of 3024 participants from 59 countries who submitted the questionnaire between November 2020 and January 2021, 2964 (98.01%) provided information that enabled the proper analysis of the results (Table 1). The mean age of the participants was 33.2 years (SD: ±7.5) and the distribution of participants between the stages of endometriosis was as follows: Stage 1: 4.8% (n  =  142); Stage 2: 9% (n  =  267); Stage 3: 14.7% (n  =  435); and Stage 4: 30.7% (n  =  910), as indicated in Table 1. In total, 40.8% (n  =  1210) of participants stated that they were not currently diagnosed with a specific stage of the disease.

In Table 2, responders were divided into four groups (A-D) according to the decreasing extent to which symptoms affected their lives (*p* << 0.001). The resulting groups were compared based on stages of endometriosis, as seen in Table 2 and Figure 1. A total of 97.77% (n = 2956) of participants provided information that enabled the analysis of the results for this section. Of these participants, 92% reported an effect on their quality of life (n = 2734). Analysis showed that the impact of endometriosis on everyday life is correlated to its stage, as shown in Figure 1 (*p*-value << 0.001.) Groups A (severe long-term effect) and B (severe short- term effect), were the most highly impacted groups, respectively. The incidence of severe effect on quality of life increases in proportion to the diagnostic stage. This is in contrast to groups C (mild effect) and D (no effect), where there is an inverse proportion to the diagnostic changes.

Table 3 and Figure 2 show that there is a complex correlation between pain experience and the diagnosed stage of endometriosis (*p*: <<0.001). A total of 95.55% (n = 2889) of participants provided information that enabled an analysis of the results.

Stage 1 was the stage most associated with pain related to triggering factors (6.6%) and least associated with chronic pain throughout the entire menstrual cycle (16.2%). It was also the stage with the largest number of respondents reporting little to no experience of pain (9.6%). Chronic pain throughout the entire menstrual cycle was reported with the highest extent in patients diagnosed with Stage 2 disease (32.2%). Similarly, among patients that were not yet diagnosed with a specific stage of disease (patients who did not undergo laparoscopic diagnosis), 28.8% reported pain throughout the entire menstrual cycle. Patients in Stage 3 (27.8%) and Stage 4 (27.7%) were fairly comparable in their experiences of chronic pain. Other (atypical) pain presentations were most pronounced in patients diagnosed with Stage 4 disease (11.4%). Pain related solely to sexual intercourse (dyspareunia) was the most uncommon pain presentation of endometriosis, with the highest reports being in patients with Stage 2 disease (5.0%). Group 2 (pain only around and during menstruation) is negative correlated to stage of disease, starting at 15.4% of the participants in Stage 1, continually decreasing to 6.9% of the participants in Stage 4. Group 6 (other experiences of pain) is positively correlated with stage; in other words, ‘other experiences of pain’ were more prevalent in patients with later stages of the disease (Stage 1 to Stage 4: 2.9% -> 2.7% -> 6.6% -> 11.4%).

Table 4 and Figure 3 describe how frequently patients with different pain experiences seek medical attention during the year. The correlation is highly significant with a *p*-value << 0.001. There is a positive correlation between chronic pain and the frequency of medical appointments from Stage 1 through Stage 3, yet it flattens between Stage 3 and 4. In contrast, there is a negative correlation between pain only during menstruation and frequency of visits. Patients most commonly attend a medical appointment once every six months for mixed pain presentation (47.2% n = 513). Chronic pain was found to be the predominant type of pain presentation that causes patients to seek medical attention multiple times during half of a year (36.5% n = 270). Chronic pain was the second most predominant type of pain presentation that causes multiple visits per month (34.3%), after the mixed presentation of pain (44.4%).

Table 5 and Figure 4 depict the stage of endometriosis in relation to fertility (*p*-value: <<0.001). There is a correlation between higher stages of disease and difficulty conceiving, with 23.9% of patients affected by infertility in Stage 1 of the disease, growing to 36.4% in Stage 4. The greatest variation exists between Stage 2 (25.5%) and Stage 3 (34.7%), while no remarkable difference in fertility hardship is seen between Stage 3 and Stage 4 (34.7% and 36.4%, respectively). As the purpose of this analysis was to identify fertility hardship, it did not include the analysis of patients reporting that they had not been trying to conceive or are not currently trying to conceive.

Table 6 and Figure 5 show the comparison between the observed distribution of pain minus the expected distribution of pain to the particular stage of endometriosis. Positive or negative results demonstrate a category of pain that is overrepresented or underrepresented within that stage, respectively.

## 4. Discussion

This study portrays the symptomatology of endometriosis as described by women suffering from the disease and its effects on their quality of life. According to priorities established following the World Congress on Endometriosis, further research should collect and evaluate data across populations and specifically focus on the symptomatology of the disease with the attempt to finalize characterization beyond the staging system that is currently used [12]. This study has therefore drawn data from 2964 participants from 59 countries who are diverse in terms of age, nationality, and country of residence, and hence provided the opportunity to present a well-established estimation accounting for the symptomatic manifestation of their disease.

Despite the availability of medical care and mental support for people with endometriosis in many countries, 92% of the women reported an effect on their quality of life. Of those, more than half (50.7%)] reported this effect to be severe in both the short and long-term. 75.1% reported that the severe effect on their quality of life was long-term. Various factors may influence the quality of life, but these findings suggest a need for improved treatment and follow-up across all stages, as well as the need to prioritize the long-term quality of life in patients living with endometriosis.

A study [13], which investigated the impact of endometriosis on quality of life and mental health, found that endometriosis with pelvic pain was associated with a lower quality of life. This is supported by the results of the present study, as it shows that women diagnosed with higher stages than Stage 1 suffered more often from chronic pain throughout the menstrual cycle, as well as having severe long term effects to their quality of life.

Nearly half of the patients (44.7%) that were not yet diagnosed with a specific stage of disease describe severe long- and short-term effects on their quality of life, which may indicate that a considerable amount of the patients who are undiagnosed suffer from advanced stage endometriosis. For the purpose of comparison, only about a third of the patients suffering from stage 1 (30.5%) reported these severe effects on their quality of life, as compared to the reports of 45.7% of patients in Stage 3. As such, undiagnosed patients seem to have results more consistent with patients in later stages of the disease, namely Stages 2 and 3.

It is possible that the large incidence of undiagnosed patients noticing severe effects on their quality of life could be the result of a negative impact on their mental and physical state due to diagnostic delay. A negative effect on health-related quality of life and its relationship with diagnostic delays was discussed in several studies. Among those, a multinational study that included 1418 premenopausal women undergoing laparoscopy demonstrated that health-related quality of life scores were lower among those who experienced a longer diagnostic delay [1]. There is a critical need to prevent diagnostic delay in patients with endometriosis in order to provide treatment as soon as possible. In order to do so, the barriers to diagnosis need to be studied. Common barriers that have been described in the literature include a lack of knowledge and awareness of the disease, the absence of noninvasive diagnostics, limitations of current treatment options, difficulties in accessibility, as well as the broader difficulties caused by the normalization of women’s pain compounded by the stigma around menstruation [14]. Addressing these barriers is an important step to increasing access to appropriate treatment.

The pathophysiology of pain in endometriosis is still unclear, as a complex interaction between physical and psychosocial factors is suggested [15,16,17]. Interestingly, previous literature claims that different types of pain, as well as different symptoms related to endometriosis, are poor predictors of its stage. No clear correlation between the stage of endometriosis and the occurrence or severity of pain symptoms was established in accordance with other studies reporting similar pain characteristics [18]. This study, therefore, also intended to examine if specific characteristics and the timing of pain can help to differentiate between stages, and thus serve as a diagnostic tool.

A mixed presentation of when pain occurred (throughout the menstrual cycle, around menses, during intercourse, or with other triggers) was the most common pain manifestation. The resulting data are in accordance with other researchers who reported that endometriosis commonly presents with a variety of pain characteristics. In this view, clinicians with the responsibility to diagnose and treat endometriosis should be aware of the possibility of multiple pain features presenting simultaneously. For instance, patients may report that at times pain appears only around or during menstruation, while others may report pain that is constantly present. Specific symptoms, such as dyspareunia or limitation in daily activities should also be considered, as well as the general effect of pain symptoms on patients’ well-being [19].

Nevertheless, our results reveal that the occurrence of pain episodes and their frequency appear to be a major factor to consider when suspecting an alteration in patients’ disease course. In our study, it was evident that patients suffering from stage 1 endometriosis experienced less constant pain throughout the menstrual cycle (16.2%), as compared with those diagnosed with other stages. This is notably lower than that which was reported by patients diagnosed with other stages, with those with Stage 3 and 4 disease having an incidence of 27.8% and 27.7%, respectively, and those in stage 2 having an incidence of 32.2%. These findings suggest that a patient is more likely to report chronic pain if they are diagnosed at Stage 2 or higher.

The prevalence of pain only around and during menstruation was reported in stage 1 significantly more than in the other stages. In fact, this pain manifestation was shown to be remarkably underrepresented among patients suffering from stage 4 disease (Difference Value = −20.883).

The findings, which show that patients in Stage 4 have the highest incidence of special pain characteristics, are of high clinical value due to the fact that such patients may be less likely to present with common pain symptoms of endometriosis. In addition, future research must be corrected to include length of pain episodes as well as more detailed characteristics, rather than focusing only on pain presence and intensity.

Pain related solely to triggering factors was the lowest reported pain manifestation, with an incidence of 3% of all patients, a finding that can also serve as a tool under the clinical interview of a patient with a suspicion of endometriosis. Triggering factors may include pain only during sexual intercourse or pain only during menstruation or ovulation. As such, complaints may be indicative of other conditions such as pelvic adhesions, adenomyosis, and gastrointestinal or urologic disorders, and thus a differential diagnosis is important and should be carefully considered as a gold standard [3].

As endometriosis is a progressive disease in many cases, symptomatic observations such as those described above are essential [20]. Previous literature suggests that early diagnosis and treatment of endometriosis may contribute to a better disease prognosis. For example, removal of lesions in the early stages of progression in animal models reversed the pain experienced [21,22], whilst removing them at later stages had no effect [22]. As laparoscopy is not considered practical as a first line diagnostic tool [3], the identification of alarming symptomatic transformation and a proper clinical interview is of high importance, and future research should address this matter further. Moreover, a delay in diagnosis may also lead to a decline in reproductive potential and fertility that is exacerbated based on how delayed a patient is in their diagnosis [3].

Understanding the relationship between endometriosis and fertility is challenging. Our findings are consistent with the previous literature, which states that the more severe the stage is, the more difficult it is for a patient to conceive [23]. Regardless, almost one quarter of patients that are not diagnosed with a specific stage of endometriosis reported difficulties in conceiving. The literature regarding infertility outcomes in relation to endometriosis stage is inconsistent. According to the recommendations of the European Society of Human Reproduction and Embryology, patients diagnosed with advanced stages (stage 3 and 4) should be referred to a reproductive medicine specialist as soon as the decision to conceive is made, due to reduced fertility rates and a prolonged conception waiting period [18]. Other sources, however, claim that the ASRM and other classification systems have poor correlation with fertility outcomes [24].

Bearing these challenges in mind, undiagnosed patients who most likely suffer from an advanced stage based on other findings might significantly benefit from early referral to fertility treatments.

## 5. Limitations

While the present study was designed to minimize limitations, some did arise. Firstly, issues regarding cultural differences as well as subjective answers are likely inevitable with an international questionnaire. The research team ensured, in most cases, to incorporate two people who spoke the targeted language during the translation. However, the team could not always ensure that two translators with English as their mother tongue were also fluent in the target language in the backward translation.

Furthermore, the questionnaire was anonymous, and was not monitored for multiple responses from a singular participant. However, there is little reason to suspect otherwise, as no incentive to participate was given. The same might apply for the diagnostic credibility of the participants. This does, however, not exclude the possibility of self-selection bias among the responders. The context of the study did not permit the verification of self-reported histological data; however, there was no incentive for the participants to misrepresent their answers. Moreover, as an endometriosis diagnosis is conducted differently around the world, the questionnaire did not investigate the means of diagnosis in depth.

With the help of national and international endometriosis organizations, the distribution of the survey through various platforms led to varying levels of success, and in some countries the questionnaire was not released at all. South America and Europe were the most represented areas, comprising 90% of the respondents.

Finally, the study surveyed a large population size of women affected by endometriosis; however, it lacks a control group. Future studies should compare symptoms in women with and without endometriosis to assess the incidence of pain and related symptoms in the general population and in the studied populations.

## 6. Conclusions

Endometriosis is a chronic disease that can lead to a significant decrease in quality of life, namely in terms of pain and fertility. Therefore, it is imperative to further understand how its clinical presentation relates to its surgical staging. The diagnostic lag time of endometriosis must be shortened in order to provide patients with proper and suitable treatment options prior to disease progression and/or symptomatic deterioration. Although endometriosis presents with a variety of symptoms, in this study we demonstrated that the characteristics of pain and quality of life tend to differ depending on the stage of the disease. Previous studies focused mainly on where and how pain is felt. Our aim was to investigate the timing of pain and how it affects quality of life. It is our belief that differentiating between the nuances of the timing of symptoms experienced provides more clinical context to identify the most probable corresponding stage of endometriosis when laparoscopy is not easily accessible or desired, which can therefore lead to a more accurate diagnostic evaluation and subsequent choice of treatment. This could lead to a shorter diagnostic lag and earlier treatment that is tailored to the patients’ needs, improving quality of life and fertility for those with endometriosis [25].

## Figures and Tables

**Table 1:** Demographic and clinical characteristics of women with endometriosis who completed the survey.

Variable		N/mean	SD/percentage	Min-Max	CI 95
Age (years)		33.2	7.5	12–72	32.9–33.5
Age at diagnosis (years)		27.7	-	-	-
Endometriosis stage	Stage 1	142	4.8%	-	-
	Stage 2	267	9%	-	-
	Stage 3	435	14.7%	-	-
	Stage 4	910	30.7%	-	-
	Unknown	1210	40.8%	-	-
How often do you seek treatment?	Once a year	668	22.5%	-	-
	Once every 6 months	1112	37.5%	-	-
	Multiple times during a half year	762	25.7%	-	-
	Multiple times a month	103	3.5%	-	-
	Other	319	10.8%	-	-

**Table 2:** Everyday experiences of quality of life by participants in relation to stage at time of completing the questionnaire.

Everyday Experience	Stage	n	%	Text	n tot.
Group A—Severe long-term effect on quality of life	Stage 1 (N = 141)	34	24.11347518	24.1%	1042
	Stage 2 (N = 267)	93	34.83146067	34.8%	
	Stage 3 (N = 435)	150	34.48275862	34.5%	
	Stage 4 (N = 907)	385	42.44762955	42.4%	
	Undiagnosed (N = 1206)	380	31.50912106	31.5%	
Group B—Severe short-term effect on quality of life	Stage 1 (N = 141)	9	6.382978723	6.4%	345
	Stage 2 (N = 267)	23	8.61423221	8.6%	
	Stage 3 (N = 435)	49	11.26436782	11.3%	
	Stage 4 (N = 907)	105	11.57662624	11.6%	
	Undiagnosed (N = 1206)	159	13.1840796	13.2%	
Group C—Mild effect on quality of life	Stage 1 (N = 141)	78	55.31914894	55.3%	1347
	Stage 2 (N = 267)	128	47.94007491	47.9%	
	Stage 3 (N = 435)	212	48.73563218	48.7%	
	Stage 4 (N = 907)	359	39.58103638	39.6%	
	Undiagnosed (N = 1206)	570	47.26368159	47.3%	
Group D—No effect on quality of life	Stage 1 (N = 141)	20	14.18439716	14.2%	222
	Stage 2 (N = 267)	23	8.61423221	8.6%	
	Stage 3 (N = 435)	24	5.517241379	5.5%	
	Stage 4 (N = 907)	58	6.394707828	6.4%	
	Undiagnosed (N = 1206)	97	8.043117745	8%	

**Table 3:** Pain experiences related to stage by participants at the time of completing the questionnaire.

Pain Experience	Stage	n	Tot	%	Text
Group 1—Chronic pain throughout the menstrual cycle	Stage 1 (N = 136)	22	810	16.18	16.2%
	Stage 2 (N = 261)	84		32.18	32.2%
	Stage 3 (N = 424)	118		27.83	27.8%
	Stage 4 (N = 886)	245		27.65	27.7%
	Undiagnosed (N = 1182)	341		28.85	28.8%
Group 2—Pain only around and during menstruation	Stage 1 (N = 136)	21	267	15.44	15.4%
	Stage 2 (N = 261)	27		10.34	10.3%
	Stage 3 (N = 424)	36		8.49	8.5%
	Stage 4 (N = 886)	61		6.88	6.9%
	Undiagnosed (N = 1182)	122		10.32	10.3%
Group 3—Pain related to trigger factors	Stage 1 (N = 136)	9	92	6.62	6.6%
	Stage 2 (N = 261)	7		2.68	2.7%
	Stage 3 (N = 424)	11		2.59	2.6%
	Stage 4 (N = 886)	20		2.26	2.3%
	Undiagnosed (N = 1182)	45		3.81	3.8%
Group 4—Pain related only to sexual intercourse	Stage 1 (N = 136)	3	93	2.21	2.2%
	Stage 2 (N = 261)	13		4.98	5%
	Stage 3 (N = 424)	11		2.59	2.6%
	Stage 4 (N = 886)	34		3.84	3.8%
	Undiagnosed (N = 1182)	32		2.71	2.7%
Group 5—Mixed presentation of pain	Stage 1 (N = 136)	64	1307	47.06	47.1%
	Stage 2 (N = 261)	118		45.21	45.2%
	Stage 3 (N = 424)	212		50	50%
	Stage 4 (N = 886)	388		43.79	43.8%
	Undiagnosed (N = 1182)	525		44.41	44.4%
Group 6—Other experiences of pain	Stage 1 (N = 136)	4	210	2.94	2.9%
	Stage 2 (N = 261)	7		2.68	2.7%
	Stage 3 (N = 424)	28		6.60	6.6%
	Stage 4 (N = 886)	101		11.40	11.4%
	Undiagnosed (N = 1182)	70		5.92	5.9%
Group 7—Little to no experience of pain	Stage 1 (N = 136)	13	110	9.56	9.6%
	Stage 2 (N = 261)	5		1.92	1.9%
	Stage 3 (N = 424)	8		1.89	1.9%
	Stage 4 (N = 886)	37		4.18	4.2%
	Undiagnosed (N = 1182)	47		3.98	4%

**Table 4:** Pain experiences in relation to gynecological appointments by the patients at the time of completing the questionnaire.

Pain Experience	n Visits	n	%	Text
Group 1—Chronic pain throughout the menstrual cycle	Once a year (N = 649)	140	21.57	21.6%
	Once every 6 months (N = 1088)	291	26.74	26.7%
	Multiple times during half year (N = 740)	270	36.49	36.5%
	Multiple times a month (N = 99)	34	34.34	34.3%
	Other (N = 313)	75	23.96	24%
Group 2—Pain only around and during menstruation	Once a year (N = 649)	88	13.56	13.6%
	Once every 6 months (N = 1088)	106	9.74	9.7%
	Multiple times during half year (N = 740)	41	5.54	5.5%
	Multiple times a month (N = 99)	2	2.02	2%
	Other (N = 313)	30	9.58	9.6%
Group 3—Pain related to trigger factors	Once a year (N = 649)	28	4.31	4.3%
	Once every 6 months (N = 1088)	35	3.22	3.2%
	Multiple times during half year (N = 740)	18	2.43	2.4%
	Other (N = 313)	11	3.51	3.5%
Group 4—Pain related only to sexual intercourse	Once a year (N = 649)	27	4.16	4.2%
	Once every 6 months (N = 1088)	28	2.57	2.6%
	Multiple times during half year (N = 740)	22	2.97	3%
	Multiple times a month (N = 99)	3	3.03	3%
	Other (N = 313)	13	4.15	4.2%
Group 5—Mixed presentation of pain	Once a year (N = 649)	275	42.37	42.4%
	Once every 6 months (N = 1088)	513	47.15	47.2%
	Multiple times during half year (N = 740)	343	46.35	46.4%
	Multiple times a month (N = 99)	44	44.44	44.4%
	Other (N = 313)	132	42.17	42.2%
Group 6—Other experiences of pain	Once a year (N = 649)	47	7.24	7.2%
	Once every 6 months (N = 1088)	72	6.62	6.6%
	Multiple times during half year (N = 740)	38	5.14	5.1%
	Multiple times a month (N = 99)	15	15.15	15.2%
	Other (N = 313)	38	12.14	12.1%
Group 7—Little to no experience of pain	Once a year (N = 649)	44	6.78	6.8%
	Once every 6 months (N = 1088)	43	3.95	4%
	Multiple times during half year (N = 740)	8	1.08	1.1%
	Multiple times a month (N = 99)	1	1.01	1%
	Other (N = 649)	14	4.47	4.5%

**Table 5:** Fertility abnormalities reported by participants in relation to stage at the time of completing the questionnaire.

Fertility Hardship	Stage	n	%	Text
Fertility related difficulties—Yes	Stage 1 (N = 142)	34	23.94	23.9%
	Stage 2 (N = 267)	68	25.47	25.5%
	Stage 3 (N = 435)	151	34.71	34.7%
	Stage 4 (N = 910)	331	36.37	36.4%
	Undiagnosed (N = 1210)	277	22.89	22.9%
Fertility related difficulties—No	Stage 1 (N = 142)	108	76.06	76.1%
	Stage 2 (N = 267)	199	74.53	74.5%
	Stage 3 (N = 435)	284	65.29	65.3%
	Stage 4 (N = 910)	579	63.63	63.6%
	Undiagnosed (N = 1210)	933	77.11	77.1%

**Table 6:** Observed distribution of pain minus expected distribution of pain in relation to stage by participants at the time of the completion of the questionnaire.

Pain Experience	Stage	Observed	Expected	Difference
Group 1—Chronic pain throughout the menstrual cycle	Stage 1 (N = 136)	22	38.13	−16.13
	Stage 2 (N = 261)	84	73.178	10.82
	Stage 3 (N = 424)	118	118.88	−0.88
	Stage 4 (N = 886)	245	248.41	−3.41
	Undiagnosed (N = 1182)	341	331.40	9.60
Group 2—Pain only around and during menstruation	Stage 1 (N = 136)	21	12.57	8.43
	Stage 2 (N = 261)	27	24.12	2.88
	Stage 3 (N = 424)	36	39.19	−3.19
	Stage 4 (N = 886)	61	81.88	−20.88
	Undiagnosed (N = 1182)	122	109.24	12.76
Group 3—Pain related to trigger factors	Stage 1 (N = 136)	9	4.33	4.67
	Stage 2 (N = 261)	7	8.31	−1.31
	Stage 3 (N = 424)	11	13.50	−2.50
	Stage 4 (N = 886)	20	28.21	−8.21
	Undiagnosed (N = 1182)	45	37.64	7.36
Group 4—Pain related only to sexual intercourse	Stage 1 (N = 136)	3	4.38	−1.38
	Stage 2 (N = 261)	13	8.40	4.60
	Stage 3 (N = 424)	11	13.65	−2.65
	Stage 4 (N = 886)	34	28.52	5.48
	Undiagnosed (N = 1182)	32	38.05	−6.05
Group 5—Mixed presentation of pain	Stage 1 (N = 136)	64	61.53	2.47
	Stage 2 (N = 261)	118	118.08	−0.08
	Stage 3 (N = 424)	212	191.82	20.18
	Stage 4 (N = 886)	388	400.83	−12.83
	Undiagnosed (N = 1182)	525	534.74	−9.74
Group 6—Other experiences of pain	Stage 1 (N = 136)	4	9.89	−5.89
	Stage 2 (N = 261)	7	18.98	−11.97
	Stage 3 (N = 424)	28	30.82	−2.82
	Stage 4 (N = 886)	101	64.40	36.60
	Undiagnosed (N = 1182)	70	85.92	−15.92
Group 7—Little to no experience of pain	Stage 1 (N = 136)	13	5.18	7.82
	Stage 2 (N = 261)	5	9.94	−4.94
	Stage 3 (N = 424)	8	16.14	−8.14
	Stage 4 (N = 886)	37	33.73	3.27
	Undiagnosed (N = 1182)	47	45.01	1.99

## Data Availability

The data underlying this article will be shared upon reasonable request to the corresponding author.
